# Bayesian hierarchical clustering for microarray time series data with replicates and outlier measurements

**DOI:** 10.1186/1471-2105-12-399

**Published:** 2011-10-13

**Authors:** Emma J Cooke, Richard S Savage, Paul DW Kirk, Robert Darkins, David L Wild

**Affiliations:** 1 Department of Chemistry, University of Warwick, Coventry, UK; 2 Systems Biology Centre, University of Warwick, Coventry, UK

## Abstract

**Background:**

Post-genomic molecular biology has resulted in an explosion of data, providing measurements for large numbers of genes, proteins and metabolites. Time series experiments have become increasingly common, necessitating the development of novel analysis tools that capture the resulting data structure. Outlier measurements at one or more time points present a significant challenge, while potentially valuable replicate information is often ignored by existing techniques.

**Results:**

We present a generative model-based Bayesian hierarchical clustering algorithm for microarray time series that employs Gaussian process regression to capture the structure of the data. By using a mixture model likelihood, our method permits a small proportion of the data to be modelled as outlier measurements, and adopts an empirical Bayes approach which uses replicate observations to inform a prior distribution of the noise variance. The method automatically learns the optimum number of clusters and can incorporate non-uniformly sampled time points. Using a wide variety of experimental data sets, we show that our algorithm consistently yields higher quality and more biologically meaningful clusters than current state-of-the-art methodologies. We highlight the importance of modelling outlier values by demonstrating that noisy genes can be grouped with other genes of similar biological function. We demonstrate the importance of including replicate information, which we find enables the discrimination of additional distinct expression profiles.

**Conclusions:**

By incorporating outlier measurements and replicate values, this clustering algorithm for time series microarray data provides a step towards a better treatment of the noise inherent in measurements from high-throughput genomic technologies. Timeseries BHC is available as part of the R package 'BHC' (version 1.5), which is available for download from Bioconductor (version 2.9 and above) via http://www.bioconductor.org/packages/release/bioc/html/BHC.html?pagewanted=all.

## Background

Post-genomic molecular biology has resulted in an explosion of typically high dimensional, structured data from new technologies for transcriptomics, proteomics and metabolomics. Often this data measures readouts from large sets of genes, proteins or metabolites over a time course rather than at a single time point. Most biological time series aim to capture information about processes which vary over time, and temporal changes in the transcription program are often apparent [1].

Grouping together genes which exhibit similar variations in expression over time can identify genes that are likely to be co-regulated by the same transcription factors [2]. Whilst there are many clustering algorithms available which allow genes to be grouped according to changes in expression level, the standard approaches to clustering use pairwise similarity measures, such as correlation or Euclidean distance, to cluster genes on the basis of their expression pattern. These algorithms disregard temporal information: the implicit assumption is that the observations for each gene are independent and identically distributed (*iid*) and are invariant with respect to the order of the observations. If the order of observations in two sequences is permuted, their correlation or Euclidean distance will not change. However, this does not hold for time series, where each observation depends on its past, and gene expression levels at adjacent time points exhibit correlation. This was demonstrated in the classic paper of Eisen *et al*. [2], who observed that the biologically meaningful clusters obtained by hierarchical clustering of *S. cerevisiae *microarray time series data, using a correlation distance metric, disappeared when the observations within each sequence were randomly permuted.

McLachlan *et al*. [3] use a model-based approach to clustering microarray data, and demonstrate the clustering of a relatively small number of tissue samples on a very large number of genes. Model-based approaches to time series clustering have included the use of finite and infinite hidden Markov models [4,5]. Another popular approach is the use of splines as basis functions [6-9]. Liverani *et al*. [10] also use Fourier series as basis functions. Ng *et al*. [11] use a random-effects model for mixture model-based clustering of correlated microarray data, including gene profiles over time. A number of additional methods for time series data analysis have been reviewed by Bar-Joseph [12].

The Bayesian Hierarchical Clustering (BHC) algorithm [13] is a fast approximate inference method for a Dirichlet process mixture model, which performs agglomerative hierarchical clustering in a Bayesian framework. BHC has previously been used to cluster genes from single time point microarray observations [14]. Heard [15], has applied an iterative reclassification extension to BHC which leads to improvements in the quality of the clustering. In this paper we extend BHC for use with time series data. Microarray time series data sets often contain several replicate values per observation and standard clustering algorithms lack the ability to incorporate this information, two exceptions being the methods of Ng *et al*. [11] and Zhou *et al*. [16]. Ng *et al*. [11] demonstrate an extension of finite mixture model clustering by introducing random gene effects and random tissue effects, such that within each cluster the random gene effects are shared among replicate measurements from the same gene (in the same tissue), while random tissue effects are shared among measurements from the same tissue. In their Bayesian model-based approach, Zhou *et al*. [16] use the information from replicate experiments to inform prior distributions for the data being clustered. Whereas Zhou *et al*. [16] use a replicate experiment to inform the prior distributions and then cluster single (non-replicated) observations, we adopt an empirical Bayes approach that uses all the replicate information to inform the prior distributions, and then cluster the mean of the data profiles.

Measurement error is not the only source of noise to consider. Genes regulated by the same transcription factor(s) are unlikely to have identical expression profiles for the duration of the time series, which leads to inherent variation in the expression data of co-regulated genes. Liu *et al*. [17] highlight the uncertainty about the precise biological time at which gene expression measurements are taken. Smith *et al*. [18] address the issue of outlier profiles in a data set by demonstrating a method of setting the model hyperparameters which can prevent agglomerative clustering methods such as that of Heard *et al*. [8] from combining outlier profiles into a single cluster at an early stage in the clustering. In this paper, we model the total noise variance as a sum of the measurement error variance and the inherent biological variation within a cluster. Typically, the noise inherent in gene expression microarray data is modelled with a Gaussian distribution, which provides a good model for the majority of data. However, a subset of the data may contain much higher levels of noise, which cannot be correctly modelled by the same distribution as that used for the majority of the data. By using a mixture model likelihood, we explicitly model a small proportion of the data as outlier measurements, and therefore allow genes which have noisy observations to participate in the clustering assignment, instead of being assigned to noisy and biologically meaningless clusters.

## Methods

### Bayesian Hierarchical Clustering

Agglomerative hierarchical clustering is a commonly used approach to group genes according to their expression levels. In this algorithm, each gene begins in its own cluster and at each stage the two most similar clusters are merged.

The BHC algorithm [13] performs agglomerative hierarchical clustering in a Bayesian setting. It uses a model-based criterion to decide which clusters to merge at each stage, and learns the most likely number of clusters, given the data. Another interpretation of the BHC algorithm is as a fast approximate inference method for a Dirichlet process mixture (DPM) model. DPM models are frequently used in clustering, and allow for an infinite number of clusters to be considered, although only a finite number of clusters are actually ever used to describe any data set.

The prior probability, *π*_*k*_, that a given pair of clusters, *C*_1 _and *C*_2_, should be merged is defined by the DPM and is determined solely by the concentration hyperparameter for the DPM and the number of genes currently in each partition of the clustering (see Savage *et al*. [14] for details). BHC uses Bayes' rule to find the posterior probability, *r*_*k*_, that the pair of clusters should be merged.

(1) $$r_{k}=\frac{\pi_{k}P\left(y|H_{1}^{k}\right)}{P\left(y|T_{k}\right)}$$

where ***y ***= {*y*_1_,..., *y*_*N*_} is the set of *N *data points contained in clusters *C*_1 _and *C*_2_. $P\left(y|H_{1}^{k}\right)$ is the marginal likelihood of the data given the hypothesis, $H_{1}^{k}$, that the data ***y ***belong to a single cluster and requires the specification of a likelihood function, *f*, as the probabilistic model generating the observed data, ***y***. *P*(***y***|*T*_*k*_) is the probability that the data could be partitioned in any way which is consistent with the order of assembly of the current partition, (see Heller and Ghahramani [13] for further details), and is defined recursively:

(2) $$\begin{array}{c} P\left(y|T_{k}\right)=\\ \pi_{k}P\left(y|H_{1}^{k}\right)+\left(1-\pi_{k}\right)P\left(y|T_{i}\right)P\left(y|T_{j}\right) \end{array}$$

where *T*_*i *_and *T*_*j *_are previously merged clusters containing subsets of the data in ***y***.

While *r*_*k *_is greater than 0.5, it is more likely that the data points contained in the clusters *C*_1 _and *C*_2 _were generated from the same underlying function, *f*, than that the data points should belong to two or more clusters. When *r*_*k *_is less than 0.5 for all remaining pairs of clusters, the number of clusters and partition best described by the data has been found.

### Gaussian Process Regression

Gaussian process regression (GPR) is a non-linear regression method with several previous applications in the analysis of gene expression data [1,17,19,20].

In our GPR model a single observation at time point *t*_*i *_is represented as *y*(*t*_*i*_) = *f*(*t*_*i*_) + *ε*. For each cluster, we assume the latent function ***f ***is drawn from an infinite dimensional Gaussian distribution, where the correlation structure between the points is determined by a covariance function, Σ, with hyperparameters, ***θ***_Σ_. We assume *ε *is *iid *noise with a Gaussian distribution, $N\left(0,\sigma_{\varepsilon }^{2}\right)$.

Let ***y ***= [*y*_*1,T *_. . . *y_G_,_T_*] be the *N = G *× *T *observations in a cluster of *G *genes, where the {*y*_*g*_, *_T_*} are time series of {1,..., *T*} time points. Each gene is normalised to have mean 0 and standard deviation 1 across time points. The prior of ***f ***is given for fixed values of ***θ***_Σ_, such that *P*(***f***|***θ***_Σ_) = *N *(**0**, Σ). It follows that the likelihood function for ***f ***is $P\left(y|f,\sigma_{\varepsilon }^{2}\right)=N\left(f,\sigma_{\varepsilon }^{2}I\right)$, where *I *is the *N *× *N *identity matrix. The marginal likelihood of the data, ***y***, is then:

(3) P(y|θΣ,σε2)=N(0,Σ+σε2I)=(2π)-N2|K|-12 exp(-12yT(K)-1y)

where $K=\Sigma +\sigma_{\varepsilon }^{2}I$ is the covariance function for ***y***. We have implemented both the squared exponential and cubic spline covariance functions into BHC. The probability *P *(***y***) is given for fixed ***θ***_Σ _and $\sigma_{\varepsilon }^{2}$, since all observations in a cluster are assumed to have the same latent function ***f ***and noise variance.

### Covariance Functions

The covariance function *K *describes the relationship between the values of the function, *f*, at different time points and must be positive semi-definite to be valid. In BHC we have implemented the squared-exponential covariance function *K*_*SE*_, which is a widely-used choice for *K*:

(4) $$K_{SE}\left(t_{i},t_{j}\right)=\sigma_{f}^{2}\left[\exp\left(-\frac{{\left(t_{i}-t_{j}\right)}^{2}}{2l^{2}}\right)\right]+\sigma_{\varepsilon }^{2}\delta_{ij}$$

where *δ*_*ij *_is the Kronecker delta function and *t*_*i *_and *t*_*j *_are two time points for *f*. The covariance function encodes our assumptions about the underlying signal in the data. For example in *K*_*SE *_the hyperparameter $\sigma_{f}^{2}$ is the signal variance, $\sigma_{\varepsilon }^{2}$ is the noise variance, and the length scale, *l*, is intuitively how far along the input time axis must be travelled between stationary points. As the inputs become closer in time, the value of *K*_*SE *_increases and tends to unity, meaning these values of *f *are more closely correlated. This encodes the intuition that our time series are smoothly-varying, once we have accounted for noise. We have also implemented the cubic spline covariance function, *K*_*CS*_, to facilitate comparison with the clustering method of Heard *et al*. [7], which can use cubic splines as basis functions:

(5) $$K_{CS}\left(t_{i},t_{j}\right)=\sigma_{f}^{2}\left[\frac{|t_{i}-t_{j}|\upsilon^{2}}{2}+\frac{\upsilon^{3}}{3}\right]+\sigma_{\varepsilon }^{2}\delta_{ij}$$

where *v = *min (*t*_*i*_,*t*_j_). *K*_*CS *_only has two hyperparameters, $\sigma_{f}^{2}$ and $\sigma_{\varepsilon }^{2}$, as described above, but no length hyperparameter.

### Using replicate data to learn the noise hyperparameter

For each cluster, we learn the hyperparameters ***θ***_Σ _and $\sigma_{\varepsilon }^{2}$, which maximise the marginal likelihood of the data, ***y***, using a gradient ascent method. We want to use the replicate information to inform the value of $\sigma_{\varepsilon }^{2}$. For this hyperparameter we assume a Gamma prior, where $P\left(\sigma_{\varepsilon }^{2}\right)=\text{Ga}\left(\alpha ,\beta \right)$.

The total noise variance, $\sigma_{\varepsilon }^{2}$, is assumed to be a sum of the measurement error variance, $\sigma_{m}^{2}$, and of the inherent biological variation in a cluster. We use the replicate values to calculate an estimate of the measurement error variance as follows:

(6) $$\sigma_{m}^{2}=\frac{1}{R\left(GRT-1\right)}\overset{T}{\underset{t=1}{\sum }}\overset{G}{\underset{g=1}{\sum }}\overset{R}{\underset{r=1}{\sum }}{\left(y_{r,g,t}-{ȳ}_{g,t}\right)}^{2}$$

where *G *is number of genes in the cluster, *R *is number of replicates per observation, *T *is number of time points in the time series and ${ȳ}_{g,t}=\sum_{r=1}^{R}y_{r,g,t}∕R$, where {*y*_*r,g,t*_} is the set of replicates for an observation.

It is these averages of the replicate values, $\left\{{ȳ}_{g,t}\right\}$, that are used as the observations in the clustering algorithm.

**Figure 1:**
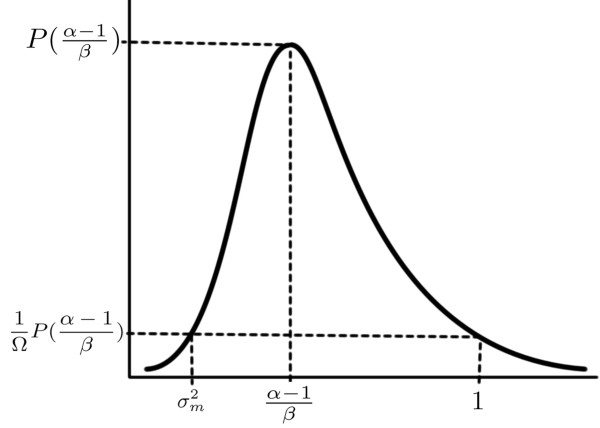
**Gamma prior on the total noise variance**. A Gamma prior is assumed for the hyperparameter $\sigma_{\varepsilon }^{2}$. This reflects our prior knowledge that $\sigma_{m}^{2}$ is a lower bound for the total noise variance. The total noise variance is unlikely to be greater than the total variance of the data, which is approximately unity because of normalisation, see Equation 7.

*P *((*α *- 1)/*β*) is the modal value of the Gamma distribution, and the hyperparameters *α *and *β *are chosen to give a weakly informative prior on $\sigma_{\varepsilon }^{2}$ such that:

(7) $$\frac{1}{\Omega }P\left(\frac{\alpha -1}{\beta }\right)\approx P\left(1\right)\approx P\left(\sigma_{m}^{2}\right)$$

where *P *denotes the Gamma distribution and Ω is chosen to be 100. Equation 7 reflects our prior knowledge that $\sigma_{m}^{2}$ is a lower bound for the total noise variance, and also that the total noise variance is unlikely to be greater than the total variance of the data, which is approximately unity because of initial normalisation, see Figure 1.

The hyperparameters, $\theta =\left(\theta_{\Sigma },\sigma_{\varepsilon }^{2}\right)$, are estimated by maximising log *P *(***θ***|***y***) using a gradient ascent method. The partial gradient of the log marginal likelihood with respect to $\theta_{j}=\sigma_{\varepsilon }^{2}$ is:

(8) ∂∂θj logP(θ|y)=12trγγT-K-1∂K∂θj+αj-1θj-βj

where *γ *= *K*^-1^*y, ∂ K*/*∂θ*_*j *_is a matrix of element-wise derivatives and '*tr*' denotes the trace of the matrix. In the case of the remaining hyperparameters, a flat prior, *P *(*θ*_*j*_), is assumed, and therefore the corresponding partial gradients contain only the trace term above. If replicate information is not required to be included in BHC, a flat prior is also assumed for $\sigma_{\varepsilon }^{2}$.

### Modelling outliers

We have so far considered the total noise in microarray measurements to have a Gaussian distribution. However, despite averaging replicate values, microarray data typically contain some outliers that are not well modelled by the Gaussian noise distribution used for the majority of the data.

Kuss *et al*. [21] introduce the idea of a mixture model for the likelihood function, *P *(***y***|***f***), where the likelihood functions for observations with regular noise have a Gaussian distribution, and a likelihood function of a different form is assumed for the outlier measurements. Stegle *et al*. [1] used such a mixture model likelihood with an expectation propagation scheme to identify differentially expressed genes. They show that the mixture model likelihood provides more accurate predictions.

We simplify our notation to denote, ${ȳ}_{g,t}$, a single expression value from gene *g *and time point *t*, as *y*_*n*_. Following the reasoning in Kuss *et al*. [21], we assume there is a small probability, *b*, that this value, *y*_*n*_, was generated by an unknown likelihood function, *P*_*o*_, producing outlier measurements, and a probability *a *= 1 - *b *that *y*_*n *_is a regular value, which was generated by a Gaussian likelihood function, *P*_*r*_. This mixture likelihood function is therefore:

(9) $$P\left(y_{n}|f_{n},\theta \right)=aP_{r}\left(y_{n}|f_{n},\theta \right)+bP_{o}\left(y_{n}|f_{n},\theta \right)$$

The expression for the marginal likelihood then becomes:

(10) $$\begin{array}{c} P\left(y|\theta \right)=\\ \int dfP\left(f|\theta \right)\overset{N}{\underset{n=1}{\prod }}\left[aP_{r}\left(y_{n}|f_{n},\theta \right)+bP_{o}\left(y_{n}|f_{n},\theta \right)\right]. \end{array}$$

Multiplying out the likelihood function product would result in 2^*N *^terms. In the case that *P*_*o *_is a conjugate distribution to *P*_*r*_, evaluation of this integral would be analytically solvable, but computationally intractable for large numbers of observations. However, if the proportion of outlier measurements is small, this series can be approximated. Making the following simplifications to notation: *A*_*n *_= *P*_*r *_(*y*_*n*_|*f*_*n*_, ***θ***) and *B*_*n *_= *P*_*o *_(*y*_*n*_|*f*_n_, ***θ***) gives:

(11) $$\begin{array}{c} P\left(y|f,\theta \right)=\overset{N}{\underset{n=1}{\prod }}\left(aA_{n}+bB_{n}\right)\\ \approx a^{N}\overset{N}{\underset{n=1}{\prod }}A_{n}+a^{N-1}b\left(\overset{N}{\underset{n=1}{\sum }}\left(B_{n}\overset{N}{\underset{n^{\prime }=1,n^{\prime }\neq n}{\prod }}An^{\prime }\right)\right) \end{array}$$

The term with coefficient *a*^*N *^represents the case where no observations are outliers. Terms with coefficient *a*^*N*-1^*b *represent the case that a single observation is an outlier.

Terms with *b*^2 ^or higher order in their coefficients represent the case that two or more observations are outliers. Since *b *is small, these terms are considered to represent events unlikely to occur and are disregarded. Our first order approximation considers every datum as an outlier; higher order approximations would incur a disproportionate computational burden.

The likelihood function for the outlier terms, *B*_*n*_, is modelled as the same constant function for all measurements, *B = *1/Range, where Range is the difference between the highest and lowest observations in the data set.

When the *A*_*n *_represent Gaussian distributions, it follows that

(12) P(y|θ)≈aN(2π)-N2|K|-12 exp-12yT(K)-1y+aN-1b∑n=1NB(2π)-N-12|K-n|-12 exp(Q).

Where $Q=-\frac{1}{2}y_{-n}^{T}{\left(K_{-n}\right)}^{-1}y_{-n},y_{-n}$ is the vector of *N *- 1 observations excluding the *n*^th ^observation and *K*_-*n *_is the corresponding covariance matrix.

After optimisation of the hyperparameters for the covariance function, *K*, the proportion of outliers, 1 - *a*, is calculated to optimise the marginal likelihood *P *(***y***|***θ***). Simplifying the notation, such that *P*(***y***|***θ***) ≈ *a*^*N*^*V*_1 _+ *a*^*N*-1^(1 - a)*V*_2_, we have *a*_max _= (1 - *N*)*V2*/*N*(V_1 _- V_2_) as the value of *a *giving the highest value for *P*(***y***|***θ***). Therefore if 0 <*a*_max _< 1, then *a = a*_max_, otherwise *a = *1.

### Datasets

For the computational experiments we have used time series data sets from four published microarray studies, which we refer to as *S. cerevisiae 1, S. cerevisiae 2, H. sapiens *and *E. coli*. The *S. cerevisiae 1 *data set uses the 17 time point cell-cycle data from Cho *et al*. [22] and contains 169 genes from eight clusters as found by the multiple data source integration method of Savage *et al*. [23]. There are no replicates for this data set.

The *S. cerevisiae 2 *data set uses the 15 time point data from Orlando *et al*. [24] for the 440 genes which were identified as periodic in the paper, and which were also identified as such by Spellman *et al*. [25] and Pramila *et al*. [26]. Two independent biological replicate samples were taken for each time point. The data from Rangel *et al*. [27] comprises two biological replicates of 34 and 10 technical replicates respectively of 58 genes from an experiment investigating how the *H*. *sapiens *Jurkat T-cell line responds to PMA and ionomycin treatment. This data is used for the *H*. *sapiens *data set. These samples were taken at 10 unequally spaced time points. The data set of Carzaniga *et al*. [28] measures the transcriptional response of *E*. *coli *K-12 cells as they are moved from 10°C to 37°C at 12 unequally spaced time points. In this data set there are three biological replicates each with two technical replicates. The genes were first ranked for differential expression using the method of Stegle *et al*. [1] and the 200 top ranked differentially expressed genes used as the *E*. *coli *data set.

### Performance metrics

When comparing BHC to other clustering methods, we are interested in identifying which method produces the most biologically meaningful clusters, and therefore use the Biological Homogeneity Index (BHI) [29] as a quality measure to reflect this. We used the R package clValid [30] to calculate the BHI scores. The BHI performance metric scores a clustering partition between 0 and 1, with higher scores assigned to more biologically homogeneous partitions with respect to a reference annotation set. For these sets we used the gene ontology (GO) annotations in the Bioconductor packages *org*.*Sc.sgd.db, hgu133plus2.db *and *org.EcK12.eg.db *for the *S. cerevisiae *1 and 2, *H. sapiens *and *E. coli *data sets respectively.

The average Pearson correlation coefficient, $\text{P}\bar{\text{C}}\text{C}$, of the expression profiles within the clusters, was used as a measure of the similarity of gene expression shapes within clusters.

The BHI and average PCC both represent mean values of a large number of pairwise similarity comparisons. For BHI, we considered whether or not pairs of (annotated) genes that have been allocated to the same cluster share GO annotations. For each such pair of genes, we thereby obtained a 1 or 0, depending on whether or not the genes do (1) or do not (0) have the same annotation. The confidence intervals for the BHI scores provided in Table 1 were determined by applying a nonparametric bootstrap (1000 iterations) to the set of all calculated 0's and 1's in each cluster in order to obtain an estimate of the standard error of the mean [31]. The confidence intervals for the average PCC were determined similarly with 100 iterations.

**Table 1:** Comparison of clustering methods using performance metrics

	#	*S. cerevisiae 1*	#	*S. cerevisiae 2*	#	*H. sapiens*	#	*E. coli*
Clustering method	clusts	$\text{P}\bar{\text{C}}\text{C}\pm \text{stdev}$	clusts	$\text{P}\bar{\text{C}}\text{C}\pm \text{stdev}$	clusts	$\text{P}\bar{\text{C}}\text{C}\pm \text{stdev}$	clusts	$\text{P}\bar{\text{C}}\text{C}\pm \text{stdev}$
BHC-SE	13	**0.68 ± 0.005**	58	**0.883 ± 0.003**	6	**0.75 ± 0.009**	24	**0.84 ± 0.003**
BHC-C	9	0.66 ± 0.004	40	0.877 ± 0.002	2	0.55 ± 0.009	15	0.80 ± 0.003
SC-linear	7	0.60 ± 0.006	40	0.881 ± 0.002	4	0.69 ± 0.009	17	0.78 ± 0.004
SC-cubic	4	0.49 ± 0.005	22	0.852 ± 0.002	2	0.44 ± 0.010	8	0.67 ± 0.004
HCL	13*	0.53 ± 0.009	58*	0.881 ± 0.002	6*	0.66 ± 0.016	24*	0.68 ± 0.006
SSClust	13*	0.60 ± 0.008	58*	0.846 ± 0.003	6*	0.69 ± 0.015	24*	0.72 ± 0.010
CAGED	2	0.42 ± 0.042	6	0.606 ± 0.003	3	0.55 ± 0.020	2	0.47 ±0.005
MCLUST	8	0.60 ± 0.004	30	0.858 ± 0.002	6	**0.75 ± 0.011**	11	0.73 ± 0.004
Zhou	13*	0.60 ± 0.008	58*	0.853 ± 0.004	6*	**0.75 ± 0.011**	24*	0.74 ± 0.006

	#	*S. cerevisiae 1*	#	*S. cerevisiae 2*	#	*H. sapiens*	#	*E. coli*
Clustering method	clusts	BHI ± stdev	clusts	BHI ± stdev	clusts	BHI ± stdev	clusts	BHI ± stdev

BHC-SE	13	0.70 ± 0.07	58	**0.57 ± 0.03**	6	0.62 ± 0.06	24	0.46 ± 0.06
BHC-C	9	**0.73 ± 0.11**	40	0.55 ± 0.03	2	**0.78 ± 0.05**	15	**0.47 ± 0.04**
SC-linear	7	0.69 ± 0.10	40	0.55 ± 0.02	4	0.66 ± 0.07	17	0.35 ± 0.03
SC-cubic	4	0.64 ± 0.02	22	0.53 ± 0.01	2	0.70 ± 0.03	8	0.32 ± 0.02
HCL	13*	0.50 ± 0.04	58*	0.56 ± 0.04	6*	0.52 ± 0.07	24*	0.44 ± 0.07
SSClust	13*	0.65 ± 0.03	58*	0.56 ± 0.02	6*	0.64 ± 0.05	24*	0.36 ± 0.03
CAGED	2	0.64 ± 0.02	6	0.52 ± 0.02	3	0.68 ± 0.04	2	0.21 ± 0.01
MCLUST	8	0.69 ± 0.02	30	0.55 ± 0.02	6	0.61 ± 0.06	11	0.47 ± 0.04
Zhou	13*	0.66 ± 0.03	58*	0.54 ± 0.02	6*	0.61 ± 0.06	24*	0.43 ± 0.07

	#	*S. cerevisiae 1*	#	*S. cerevisiae 2*	#	*H. sapiens*	#	*E. coli*
Clustering method	clusts	log marginal likelihood	clusts	log marginal likelihood	clusts	log marginal likelihood	clusts	log marginal likelihood

BHC-SE	13	**-3293**	58	**-3956**	6	**-633**	24	**-2497**
BHC-C	9	-3356	40	-4294	2	-734	15	-2622

Table 1 shows the average Pearson correlation Coefficient ($\text{P}\bar{\text{C}}\text{C}$) and BHI score of the four data sets for the different clustering algorithms. Confidence intervals represent ± one standard deviation, calculated by performing a nonparametric bootstrap. For the number of clusters in the partition (# clusts),* denotes that the number has not been optimized by the algorithm, but fixed at the number obtained for BHC with squared exponential covariance. The clustering methods are explained in the Methods Section. The table also shows the log-marginal likelihoods, log (*P*(***y***|*T*)), for BHC-SE and BHC-C. The best values for each data set are in **bold**.

Over-represented GO annotations were found using the GOstat web-based interface http://gostat.wehi.edu.au for a Benjamini and Hochberg False Discovery Rate controlled at 1%, unless otherwise stated.

## Results and Discussion

### Comparison of BHC to other clustering methods

For each of the four data sets, we compared the BHC time series algorithm using squared exponential (BHC-SE) and cubic spline (BHC-C) covariances to the clustering methods of SplineCluster [7] using both linear (SC-linear) and cubic (SC-cubic) splines, SSClust [9], CAGED [32] and the method of Zhou *et al *[16]. These methods are designed to account for the correlations between the observations in time series data. For a clear comparison with the BHC algorithm, we did not use a mixture model likelihood, or include any replicate information. We also compared BHC to Euclidean distance average linkage hierarchical clustering (HCL) as implemented in the MeV software [33], and MCLUST [34]. For these two methods the clustering partitions are invariant to permutation of the time points.

Freely available software is available for each method, and all but HCL estimate the number of clusters for a data set. However, the BIC score in SSClust generally continued to improve with an increasing number of clusters, suggesting overfitting. For the method of Zhou *et al*., we used the JAGS code (available from http://faculty.washington.edu/jonno/biometrics_code.txt) for the first order random walk model described in Zhou *et al*. [35], which allows incorporation of prior information. This method is a generalised case of the method described in Zhou *et al*. [16], which is specifically for periodic data. The JAGS implementation required the preferred number of clusters to be pre-specified. Details of the priors used for this method are available in Additional File 1. Therefore for HCL, SSClust and the method of Zhou *et al*., the number of clusters was fixed at the number obtained for BHC-SE. The CAGED algorithm was tried with all possible Markov orders allowed by its software, but a low number of clusters was always favoured, a phenomenon also found by Heard *et al*. [8]. We restricted the MCLUST clustering to models with spherical and diagonal covariance matrices, since we found that permitting full covariance matrices tended to yield poor results. When using SplineCluster, the prior precision on the coefficients was selected by maximisation of the log marginal likelihood of the clustering. Only BHC, SplineCluster and the method of Zhou *et al*. were able to incorporate the non-uniformly sampled time intervals for the *H. sapiens *and *E.coli *data sets.

**Figure 2:**
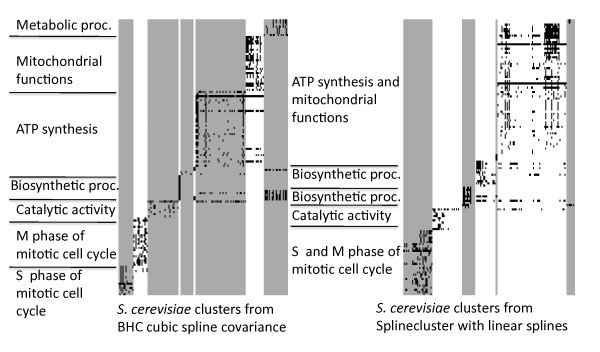
**GO annotation matrices**. Over-represented GO annotations, *p *< 0.01 for the BHC-C clusters *left *(BHI = 0.73) and the SplineCluster clusters using linear splines *right *(BHI = 0.69). The vertical grey shading separates gene clusters and each row is a GO annotation. Black shading indicates a GO annotation associated to the corresponding gene is over-represented in the cluster. A representative GO annotation is given. For the full GO annotations and a large version of the Figure, see Additional Files 3 and 4. *Data set: S. cerevisiae 1 *[22]

Table 1 shows the results of the two performance metrics $\text{P}\bar{\text{C}}\text{C}$ and BHI for these comparisons, where higher values are better for both metrics. In all cases the BHC algorithm gives the most coherent or joint most coherent clustering according to expression level, as measured by the $\text{P}\bar{\text{C}}\text{C}$. For the *H. sapiens *data set the MCLUST and Zhou methods give an identical clustering, which has an equal $\text{P}\bar{\text{C}}\text{C}$ to the almost identical clustering of the BHC-SE method. BHC also gives the most biologically relevant clustering partition as measured by the BHI, except in the case of *S. cerevisiae 1 *where the BHI confidence intervals of BHC-C and SC-linear overlap considerably. However, the greater number of clusters generated by BHC-C are more biologically meaningful (see Figure 2). Liverani *et al*. [10] also find a greater number of clusters for their data set than the SplineCluster method of Heard *et al*. [7] and demonstrate this is an improvement in the quality of clustering.

At each stage, the BHC algorithm calculates the marginal likelihood of the tree structure for the data, *p*(***y***|*T*_*k*_), as given by Equation (2). Denoting the final, unpruned tree structure returned by the algorithm as *T*, we may use the final (root node) marginal likelihood, *p*(**y**|*T*), in order to do model comparison between different choices for the covariance function (a similar strategy is employed by Heller and Ghahramani [13] in order to select model hyperparameters). In Table 1, we provide (log) marginal likelihoods for the squared exponential and cubic spline covariance functions. For all data sets considered in this paper, the squared exponential covariance function yields the higher log-marginal likelihood. We note that this is in good agreement with the $P\bar{C}C$, which is also consistently higher for BHC-SE. For all data sets the gene lists and plots of clusters for BHC-SE and BHC-C are available in Additional File 2. Figure 2 shows the over-represented GO annotations using the R package *GOstats *in the clusters resulting from BHC-C and SplineCluster using linear splines, for the *S. cerevisiae 1 *data set. Grey and white vertical shading separates the clusters and each row represents a GO annotation, where the dark block shading indicates an annotation is over-represented in the cluster. A representative GO annotation is given for each cluster. Figure 2 shows that BHC is able to separate the clusters of mitochondrial and ATP synthesis functions and also the M- and S-Phase mitotic cell cycle genes, that SplineCluster combines together. The increased biological homogeneity of the BHC clusters is reflected in a higher BHI score of 0.73, compared to a BHI for SplineCluster of 0.69. For the full GO annotations and a large version of Figure 2, see Additional Files 3 and 4.

### BHC clustering of simulated data sets

An advantage of the BHC algorithm is that it allows simulated data with realistic noise and expression profiles to be generated from the Gaussian processes inferred from the BHC clustering of real biological data.

**Figure 3:**
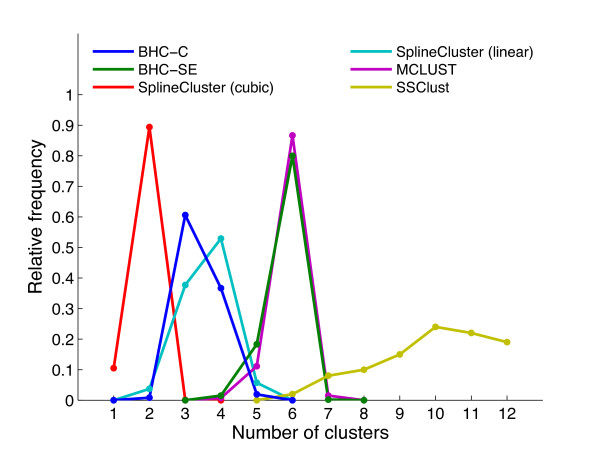
**H. sapiens simulated data**. Relative frequencies of the estimated number of clusters obtained when a variety of clustering algorithms (BHC-C, BHC-SE, SplineCluster with linear and cubic splines, MCLUST and SSClust) were applied to simulated data sets (due to slow running times, we only used 100 of the 1000 simulated data sets to obtain the SSClust results). For each clustering algorithm, we draw lines between relative frequency values to aid interpretability. Each simulated data set was generated from the 6 Gaussian processes obtained from the BHC-SE clustering of the *H. sapiens *data set, and has the same number of genes, timepoints and per cluster noise levels. Note that, for SSClust, we specified the maximum permissible number of clusters to be 12.

**Figure 4:**
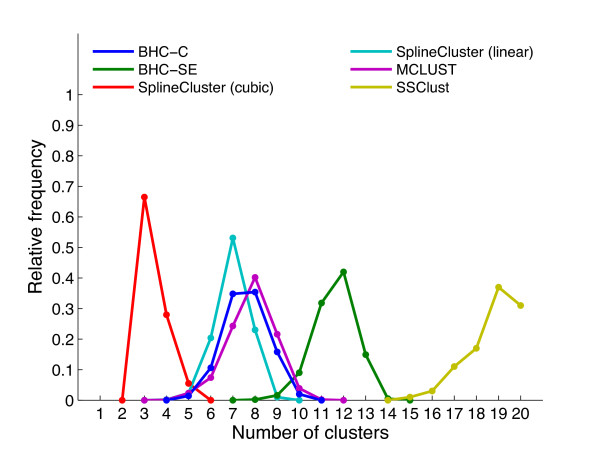
**S. cerevisiae 1 simulated data**. As Figure 3, except that simulated data sets were generated from the 13 Gaussian processes obtained from the BHC-SE clustering of the *S. cerevisiae 1 *data (again, due to slow running times, we only used 100 of our 1000 simulated data sets to obtain the SSClust results). Note that, for SSClust, we specified the maximum permissible number of clusters to be 20.

To demonstrate that the BHC algorithm can find the correct number of clusters for a synthetic data set, we analysed simulated data sets with the same number of genes, timepoints and noise levels, which were generated from the 6 and 13 Gaussian processes inferred from the BHC-SE clustering of the *H. sapiens *and *S. cerevisiae 1 *data sets respectively. These Gaussian processes are therefore models of true biological data. Figures 3 and 4 show the estimated number of clusters found for 1000 simulated *H. sapiens *and *S. cerevisiae 1 *data sets respectively, for BHC-SE, BHC-C, SplineCluster (linear and cubic), MCLUST and SSClust methods. We did not use CAGED in the comparison, since it is a Windows-based program that does not permit automation.

BHC-SE finds the correct number of clusters for the simulated data generated from the 6 Gaussian processes in 80% of cases. For the simulated data generated from the 13 Gaussian processes, BHC-SE finds between 11-13 clusters in 89% of cases. For the *H. sapiens *data, MCLUST is the only method other than BHC-SE to correctly favour 6 clusters. For the *S. cerevisiae 1 *data, BHC-SE is the only method to favour around 13 clusters. Simulated data sets generated from the Gaussian processes with half the noise standard deviation were always partitioned by BHC-SE into exactly the original number of clusters of 6 and 13 (data not shown).

### Modelling outlier measurements

**Figure 5:**
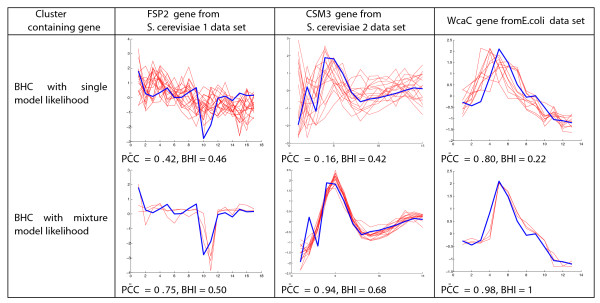
**Effect of a mixture model likelihood on noisy gene classification**. Using a mixture model likelihood allows BHC to model certain time points as outlier measurements for the genes shown, and assign the noisy gene to a cluster which is more coherent in its expression profiles and biological function. Outlier time points are time point 11 for *FSP2*, time point 2 for *CMS3 *and time point 4 for *WcaC*. The examples shown use BHC-SE for *S. cerevisiae 1 *and BHC-C for *S. cerevisiae 2 *and *E.coli*.

We investigated the effect of using the mixture model likelihood. Figure 5 shows for an example of a noisy gene from three of the data sets, the clusters to which the gene was assigned using standard BHC (with a single likelihood function) and mixture BHC (with a mixture model likelihood function).

In the *S. cerevisiae 1 *data set, four of the 169 genes were assigned to different clusters using the mixture BHC-SE instead of standard BHC-SE. There was no change in the clustering partition for BHC-C. For standard BHC-SE, the *FSP2 (IsoMaltase) *gene is annotated with several GO terms which are over-represented in its cluster (top left in Figure 5). These GO terms include *glucosidase activity*, which *FSP2 *shares with three out of 20 genes, and the whole cluster has a BHI of 0.46. When using mixture BHC-SE, the *FSP2 *gene expression at time point 11 has been treated as an outlier measurement, which has resulted in the *FSP2 *gene no longer being a member of this noisy cluster where $\text{P}\bar{\text{C}}\text{C}=0.42$, but being assigned to a tighter $\left(\text{P}\bar{\text{C}}\text{C}=0.75\right)$, more biologically homogeneous cluster (bottom left in Figure 5), with a higher BHI of 0.50, and where all of the four members, *SOR1 (Sorbitol dehydrogenase), RFC2 (Replication Factor C), RMA1 (Reduced Mating A) *and *FSP2 *are annotated as being involved in *catalytic activity*. In the *S. cerevisiae 2 *data set, the standard BHC cluster containing the *CSM3 (Chromosome Segregation in Meiosis) *gene (top middle in Figure 5 ) does not contain any over-represented GO terms at a significance level of *p *< 0.01, and has a BHI of 0.42. This cluster is quite noisy, with a $\text{P}\bar{\text{C}}\text{C}$ of 0.16. Using a mixture model likelihood allows BHC to treat the *CSM3 *expression level at time point 2 as an outlier value. This allows the *CSM3 *gene to join a tighter cluster (bottom middle in Figure 5) with a $\text{P}\bar{\text{C}}\text{C}$ of 0.94, where it shares over-represented GO terms such as *mitotic sister chromatid cohesion, DNA replication *and *M phase of mitotic cell cycle*, with 9 of the 15 cluster members.

In the *E. coli *data set, the clustering partition using BHC-C has only one gene, *WcaC, (Putative colanic acid biosynthesis glycosyl transferase) *assigned to a different cluster, when comparing standard and mixture BHC. The cluster containing this gene for standard BHC-C (top right in Figure 5) has no over-represented GO terms and a BHI of 0.22. Using the mixture mode likelihood allows time point 4 of the *WcaC *expression profile to be treated as an outlier value and the gene is reassigned to a highly correlated cluster (bottom right in Figure 5) where the $\text{P}\bar{\text{C}}\text{C}=0.98$ and where *WcaC *shares several significant GO terms with two of the other three genes in the cluster, such as *lipopolysaccharide biosynthetic process*. For all data sets the gene lists and plots of clusters for both BHC-SE and BHC-C using the mixture model likelihood are available in Additional File 2.

### Inclusion of replicate information

**Figure 6:**
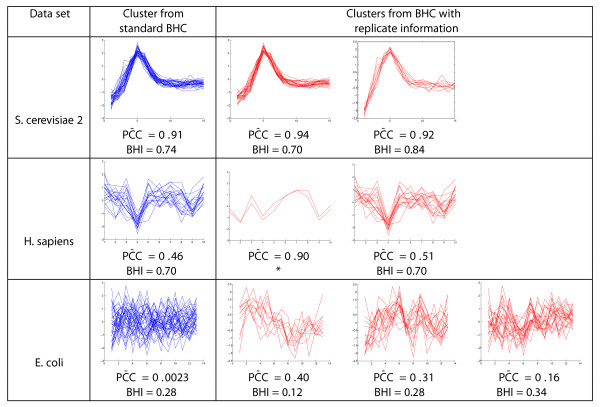
**Effect of including replicate information on noisy clusters**. Using replicate information can split a noisy cluster into smaller more biologically homogeneous clusters with distinct profiles. The examples shown use BHC-C for the *S. cerevisiae 2 *data set and BHC-SE for the *H. sapiens *and *E. coli *data sets. *For this cluster of only two genes, instead of considering the BHI, we looked directly at the biological functions of the genes.

We investigated the effect of including the replicate information. Figure 6 shows the effect on a cluster from each dataset which has replicate information.

The standard BHC cluster from the *S. cerevisiae 2 *data set (top left Figure 6) has over-represented GO terms, such as *DNA replication, DNA repair *and *hydrolase activity*. Including the replicate information in the BHC clustering resulted in profiles which are subtly different during the first few time points being distinguished and reassigned (top right Figure 6), as shown by the increase in the $\text{P}\bar{\text{C}}\text{C}$ from 0.91 to 0.92 and 0.94 for the two resulting 'child' clusters. These two child clusters have similar over-represented GO terms to the original cluster.

Including the replicate information for the *H. sapiens *data set resulted in the distinct and highly correlated $\left(\text{P}\bar{\text{C}}\text{C}=0.90\right)$, profiles of the two genes *CASP7 (Caspase 7) *and *IKZF1 (IKAROS family zinc finger 1) *being distinguished (middle row, Figure 6). These genes are both members of the disease-specific gene signature of the neoplastic disease Mantle cell lymphoma [36] and their protein products are both implicated in apoptosis [37,38].

An unusually noisy cluster (bottom left Figure 6) was formed using standard BHC for the *E. coli *data set, with a $\text{P}\bar{\text{C}}\text{C}=0.0023$. However, this cluster contains several over-represented GO terms such as *metal ion binding*. Including replicate information allows this cluster to be split into three clusters with distinct profiles (bottom right Figure 6). The child cluster with BHI = 0.12 has no over-represented GO terms and the remaining two child clusters have similar over-represented GO terms to the standard BHC cluster. For all data sets the gene lists and plots of clusters for both BHC-SE and BHC-C using replicate information are available in Additional File 2.

### Run time

Table 2 gives the run time for our BHC algorithm for each of the four data sets used in this paper. The most time-intensive calculation in the BHC algorithm is the inversion of the covariance matrix, *K*, which has dimension *GT *× *GT*, where *G *is the number of genes in a cluster and *T *is the number of time points in the data set. To reduce the calculation time, we arranged the data by order of time points, which gives the corresponding covariance matrix a block matrix structure. Using the *matrix inversion lemma *with recursion as detailed in Rasmussen [39], this then only requires the inversion of a single *T *× *T *matrix. The hyperparameter optimisations now become the factor limiting the algorithm run time.

**Table 2:** Run time

Data set	BHC-SE	BHC-SE mixture model	Genes	Time points	Replicates
*S. cerevisiae 1*	6 m 3 s	38 m 49 s	169	17	N/A
*S. cerevisiae 2*	24 m 8 s	5 h 48 m	440	15	2
*H. sapiens*	19 s	49 s	58	10	44
*E. coli*	7 m 6 s	34 m 39 s	200	13	6

Run times of data sets for BHC-SE and BHC-SE with a mixture model likelihood in hours (h), minutes (m) and seconds (s) on a 2.40 GHz Intel Xeon CPU. The run times for BHC-C were very similar to BHC-SE. Using replicate information did not increase the run times. Also shown are the number of genes, time points and replicates for each dataset.

## Conclusions

We have presented an extension to the BHC algorithm [14] for time-series microarray data, using a likelihood based on Gaussian process regression, which learns the optimum number of clusters given the data, and which incorporates non-uniformly sampled time points. We have extensively tested the performance of BHC against other leading clustering methods for four sets of time series data, and found that BHC consistently produced more coherent clusters both in terms of expression profiles and biological function.

BHC facilitates the inclusion of replicate information, and our results clearly demonstrate an improvement in the ability to distinguish between distinct expression profiles when this replicate information is included. Microarray data typically contain outlier observations, which should not be treated together with the majority of observations. One unique aspect of the BHC algorithm presented in this paper is its ability to model these noisy outlier measurements using a mixture model likelihood. The result is that genes with a small number of noisy values, which would otherwise have been assigned to a noisy cluster, are assigned to a biologically relevant cluster, where the noisy gene shares biological functions with the other cluster members. This method provides a step towards a better treatment of the noise inherent in measurements from high-throughput post-genomic technologies.

## Availability

Timeseries BHC is available as part of the R package 'BHC' (version 1.5), which is available for download from Bioconductor (version 2.9 and above) via http://www.bioconductor.org/packages/release/bioc/html/BHC.html?pagewanted=all. The timeseries functionality was developed under R version 2.13. The 'BHC' package is available for Mac OS X, Linux and Windows operating systems. 'BHC' is released under the Gnu GPL (v3).

## Authors' contributions

EJC and RSS wrote the clustering code, EJC and PDWK analysed the simulated data and performed bootstrapping, EJC performed the clustering analysis, RD optimised the C++ code and updated the BHC Bioconductor package, DLW designed and directed the research. All authors contributed ideas, participated in writing this article, and read and approved the final manuscript.

## Supplementary Material

Additional file 1 **The clustering method of Zhou et al**. Further details for running the method of Zhou *et al*. Click here for file

Additional file 2 **Genes lists and cluster plots**. Gene lists and cluster eps files for the *S. cerevisiae 1, S. cerevisiae 2, E. coli *and H. sapiens data sets using BHC with both squared exponential (BHC-SE) and cubic (BHC-C) covariances. For each covariance option, results are given for the single model likelihood, mixture model likelihood, and including replicate information. Click here for file

Additional file 3 **GO annotation matrix for S. cerevisiae 1 data set clustered using BHC with cubic spline covariance**. A large version of Figure 2, left panel. Click here for file

Additional file 4 **GO annotation matrix for S. cerevisiae 1 data set clustered using SplineCluster with linear splines**. A large version of Figure 2, right panel. Click here for file
