# Supplementary material for: Bayesian hierarchical clustering for microarray time series data with replicates and outlier measurements
Source: BMC Bioinformatics. 2011 Oct 13;12:399. doi: 10.1186/1471-2105-12-399 (PMC3228548; doi:10.1186/1471-2105-12-399)
Supplement: Additional file 1 — The clustering method of Zhou et al. Further details for running the method of Zhou et al. [file 1471-2105-12-399-S1.PDF]

1. Further details for the method of Zhou et al.

We adopted an empirical Bayes approach and used the data to inform the priors. We used the replicate data values to calculate a measurement error variance using Equation 7 to suggest a suitable region for the mode and 95% quantile of the Inverse Gamma distribution of the measurement error variance and then found parameters using numerical search. We then converted the parameters accordingly for the Gamma distribution of the measurement error precision which were input into the JAGS code. We used Gamma parameters of $(\alpha, \beta) = (3, 0.14)$ for the *H. sapiens* data set, and $(\alpha, \beta) = (3, 1)$ for the *E. coli* data set and the *S. cerevisiae 2* data set. For the *S. cerevisiae 1* data set which has no replicates we used a diffuse Gamma prior with parameters $(\alpha, \beta) = (1.27, 0.44)$. For the first time point precision and inter time point precision we used Gamma priors with parameters $(\alpha, \beta) = (1.11, 1.11)$ for all data sets which ensured the prior was diffuse around the precision value suggested by the data. We ran the MCMC chain for 200,000 iterations after a burn-in period of 100,000 iterations. We note that a C implementation of the Zhou *et al.* method is available from the authors which uses birth death MCMC to learn the number of clusters in a dataset, but this requires a commercial C library.
