# Supplementary material for: Bayesian hierarchical clustering for microarray time series data with replicates and outlier measurements
Source: BMC Bioinformatics. 2011 Oct 13;12:399. doi: 10.1186/1471-2105-12-399 (PMC3228548; doi:10.1186/1471-2105-12-399)
Supplement: Additional file 3 — GO annotation matrix for S. cerevisiae 1 data set clustered using BHC with cubic spline covariance. A large version of Figure 2, left panel. [file 1471-2105-12-399-S3.PDF]

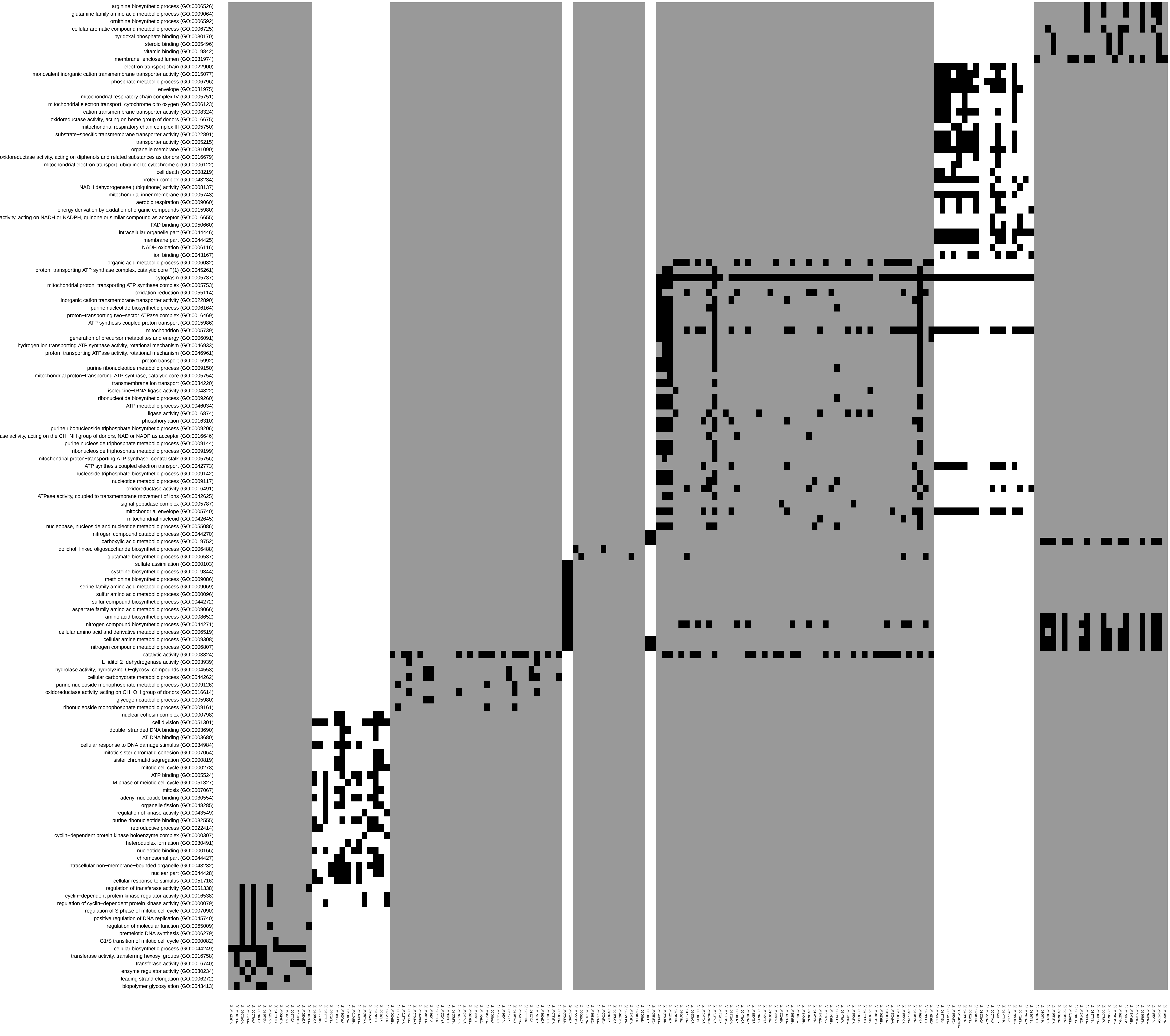

arginine biosynthetic process (GO:0006526)
glutamine family amino acid metabolic process (GO:0009064)
ornithine biosynthetic process (GO:0006592)
cellular aromatic compound metabolic process (GO:0006725)
pyridoxal phosphate binding (GO:0030170)
steroid binding (GO:0005496)
vitamin binding (GO:0019842)
membrane−enclosed lumen (GO:0031974)
electron transport chain (GO:0022900)
monovalent inorganic cation transmembrane transporter activity (GO:0015077)
phosphate metabolic process (GO:0006796)
envelope (GO:0031975)
mitochondrial respiratory chain complex IV (GO:0005751)
mitochondrial electron transport, cytochrome c to oxygen (GO:0006123)
cation transmembrane transporter activity (GO:0008324)
oxidoreductase activity, acting on heme group of donors (GO:0016675)
mitochondrial respiratory chain complex III (GO:0005750)
substrate−specific transmembrane transporter activity (GO:0022891)
transporter activity (GO:0005215)
organelle membrane (GO:0031090)
oxidoreductase activity, acting on diphenols and related substances as donors (GO:0016679)
mitochondrial electron transport, ubiquinol to cytochrome c (GO:0006122)
cell death (GO:0008219)
protein complex (GO:0043234)
NADH dehydrogenase (ubiquinone) activity (GO:0008137)
mitochondrial inner membrane (GO:0005743)
aerobic respiration (GO:0009060)
energy derivation by oxidation of organic compounds (GO:0015980)
activity, acting on NADH or NADPH, quinone or similar compound as acceptor (GO:0016655)
FAD binding (GO:0050660)
intracellular organelle part (GO:0044446)
membrane part (GO:0044425)
NADH oxidation (GO:0006116)
ion binding (GO:0043167)
organic acid metabolic process (GO:0006082)
proton−transporting ATP synthase complex, catalytic core F(1) (GO:0045261)
cytoplasm (GO:0005737)
mitochondrial proton−transporting ATP synthase complex (GO:0005753)
oxidation reduction (GO:0055114)
inorganic cation transmembrane transporter activity (GO:0022890)
purine nucleotide biosynthetic process (GO:0006164)
proton−transporting two−sector ATPase complex (GO:0016469)
ATP synthesis coupled proton transport (GO:0015986)
mitochondrion (GO:0005739)
generation of precursor metabolites and energy (GO:0006091)
hydrogen ion transporting ATP synthase activity, rotational mechanism (GO:0046933)
proton−transporting ATPase activity, rotational mechanism (GO:0046961)
proton transport (GO:0015992)
purine ribonucleotide metabolic process (GO:0009150)
mitochondrial proton−transporting ATP synthase, catalytic core (GO:0005754)
transmembrane ion transport (GO:0034220)
isoleucine−tRNA ligase activity (GO:0004822)
ribonucleotide biosynthetic process (GO:0009260)
ATP metabolic process (GO:0046034)
ligase activity (GO:0016874)
phosphorylation (GO:0016310)
purine ribonucleoside triphosphate biosynthetic process (GO:0009206)
ase activity, acting on the CH−NH group of donors, NAD or NADP as acceptor (GO:0016646)
purine nucleoside triphosphate metabolic process (GO:0009144)
ribonucleoside triphosphate metabolic process (GO:0009199)
mitochondrial proton−transporting ATP synthase, central stalk (GO:0005756)
ATP synthesis coupled electron transport (GO:0042773)
nucleoside triphosphate biosynthetic process (GO:0009142)
nucleotide metabolic process (GO:0009117)
oxidoreductase activity (GO:0016491)
ATPase activity, coupled to transmembrane movement of ions (GO:0042625)
signal peptidase complex (GO:0005787)
mitochondrial envelope (GO:0005740)
mitochondrial nucleoid (GO:0042645)
nucleobase, nucleoside and nucleotide metabolic process (GO:0055086)
nitrogen compound catabolic process (GO:0044270)
carboxylic acid metabolic process (GO:0019752)
dolichol−linked oligosaccharide biosynthetic process (GO:0006488)
glutamate biosynthetic process (GO:0006537)
sulfate assimilation (GO:0000103)
cysteine biosynthetic process (GO:0019344)
methionine biosynthetic process (GO:0009086)
serine family amino acid metabolic process (GO:0009069)
sulfur amino acid metabolic process (GO:0000096)
sulfur compound biosynthetic process (GO:0044272)
aspartate family amino acid metabolic process (GO:0009066)
amino acid biosynthetic process (GO:0008652)
nitrogen compound biosynthetic process (GO:0044271)
cellular amino acid and derivative metabolic process (GO:0006519)
cellular amine metabolic process (GO:0009308)
nitrogen compound metabolic process (GO:0006807)
catalytic activity (GO:0003824)
L−iditol 2−dehydrogenase activity (GO:0003939)
hydrolase activity, hydrolyzing O−glycosyl compounds (GO:0004553)
cellular carbohydrate metabolic process (GO:0044262)
purine nucleoside monophosphate metabolic process (GO:0009126)
oxidoreductase activity, acting on CH−OH group of donors (GO:0016614)
glycogen catabolic process (GO:0005980)
ribonucleoside monophosphate metabolic process (GO:0009161)
nuclear cohesin complex (GO:0000798)
cell division (GO:0051301)
double−stranded DNA binding (GO:0003690)
AT DNA binding (GO:0003680)
cellular response to DNA damage stimulus (GO:0034984)
mitotic sister chromatid cohesion (GO:0007064)
sister chromatid segregation (GO:0000819)
mitotic cell cycle (GO:0000278)
ATP binding (GO:0005524)
M phase of meiotic cell cycle (GO:0051327)
mitosis (GO:0007067)
adenyl nucleotide binding (GO:0030554)
organelle fission (GO:0048285)
regulation of kinase activity (GO:0043549)
purine ribonucleotide binding (GO:0032555)
reproductive process (GO:0022414)
cyclin−dependent protein kinase holoenzyme complex (GO:0000307)
heteroduplex formation (GO:0030491)
nucleotide binding (GO:0000166)
chromosomal part (GO:0044427)
intracellular non−membrane−bounded organelle (GO:0043232)
nuclear part (GO:0044428)
cellular response to stimulus (GO:0051716)
regulation of transferase activity (GO:0051338)
cyclin−dependent protein kinase regulator activity (GO:0016538)
regulation of cyclin−dependent protein kinase activity (GO:0000079)
regulation of S phase of mitotic cell cycle (GO:0007090)
positive regulation of DNA replication (GO:0045740)
regulation of molecular function (GO:0065009)
premeiotic DNA synthesis (GO:0006279)
G1/S transition of mitotic cell cycle (GO:0000082)
cellular biosynthetic process (GO:0044249)
transferase activity, transferring hexosyl groups (GO:0016758)
transferase activity (GO:0016740)
enzyme regulator activity (GO:0030234)
leading strand elongation (GO:0006272)
biopolymer glycosylation (GO:0043413)
